# Clearance of Circulating Tumor Cells in Patients with Hepatocellular Carcinoma Undergoing Surgical Resection or Liver Transplantation

**DOI:** 10.3390/cancers13102476

**Published:** 2021-05-19

**Authors:** Víctor Amado, Sandra González-Rubio, Javier Zamora, Rafael Alejandre, María Lola Espejo-Cruz, Clara Linares, Marina Sánchez-Frías, Gema García-Jurado, José Luis Montero, Rubén Ciria, Manuel Rodríguez-Perálvarez, Gustavo Ferrín, Manuel De la Mata

**Affiliations:** 1 Department of Hepatology and Liver Transplantation, Reina Sofía University Hospital, 14004 Córdoba, Spain; victoramadotorres@hotmail.com (V.A.); h22zaolj@gmail.com (J.Z.); alejandrealt@gmail.com (R.A.); jlm14005623@gmail.com (J.L.M.); mdelamatagarcia@gmail.com (M.D.l.M.); 2 Maimónides Institute of Biomedical Research (IMIBIC), University of Córdoba, 14004 Córdoba, Spain; sayuri254@hotmail.com (S.G.-R.); lolaespejo@gmail.com (M.L.E.-C.); liluc80@hotmail.com (C.L.); gema.garcia@imibic.org (G.G.-J.); rubenciria@gmail.com (R.C.); gusfesa@gmail.com (G.F.); 3 Centro de Investigación Biomédica en Red de Enfermedades hepáticas y digestivas (CIBERehd), 28029 Madrid, Spain; 4 Pathology Department, Reina Sofía University Hospital, 14004 Córdoba, Spain; marinasanchezfrias@gmail.com; 5 Department of Hepatobiliary Surgery and Liver Transplantation, Reina Sofía University Hospital, 14004 Córdoba, Spain

**Keywords:** hepatocellular carcinoma, liver transplantation, liver cancer, liquid biopsy, circulating tumor cell

## Abstract

**Simple Summary:**

Liver transplantation and surgical resection are potentially curative options in patients with liver cirrhosis and hepatocellular carcinoma. However, tumor recurrence is frequent, and it is associated with a poor prognosis. The selection of candidates is paramount to maximize survival while decreasing tumor recurrence rates, particularly regarding liver transplantation in a context of worldwide organ shortage. Circulating tumor cells are an attractive method of liquid biopsy that could represent a novel strategy to delineate the optimal therapeutic approach in hepatocellular carcinoma. This observational, prospective study aims to assess the role of circulating tumor cells in patients undergoing surgical resection or liver transplantation, as well as their potential association with other widely known surrogate markers of poor prognosis.

**Abstract:**

Background: In patients with hepatocellular carcinoma (HCC), a complete clearance of circulating tumor cells (CTCs) early after liver transplantation (LT) or surgical resection (LR) could prevent tumor recurrence. Methods: prospective pilot study including patients with HCC who underwent LR or LT from September 2017 to May 2020. Enumeration of CTCs was performed in peripheral blood samples (7 mL) using the Isoflux^®^ system (Fluxion Biosciences) immediately before surgery, at post-operative day 5 and at day 30. A clinically relevant number of CTCs was defined as >30 CTCs/sample. Results: 41 HCC patients were included (mean age 58.7 ± 6.3; 82.9% male). LR was performed in 10 patients (24.4%) and 31 patients (75.6%) underwent LT. The main etiology of liver disease was chronic hepatitis C (31.7%). Patients undergoing LR and LT were similar in terms of preoperative CTC count (*p* = 0.99), but clearance of CTCs within the first month was more pronounced in the LT group. Clusters of CTCs at baseline were associated with incomplete clearance of CTCs at day 30 (54.2% vs. 11.8%, *p* = 0.005), which in turn impacted negatively on survival (*p* = 0.038). Conclusion: Incomplete clearance of CTCs after surgery could be a surrogate marker of HCC aggressiveness.

## 1. Introduction

Hepatocellular carcinoma (HCC) is the sixth most common cancer and the fourth leading cause of cancer-related death worldwide [1]. Liver resection (LR) and liver transplantation (LT) are the first-line therapeutic options in selected patients with tumors confined to the liver [2]. LR may be used in early-stage tumors, provided that liver function is preserved, and clinically relevant portal hypertension is absent. Nevertheless, tumor recurrence may occur in up to 70% of cases [2]. On the other hand, LT is the only therapeutic option able to cure both HCC and the underlying liver cirrhosis, with 5-year survival rates above 70% in patients within Milan criteria [3]. However, tumor recurrence may occur in up to 15% of patients after LT and it is associated with a poor prognosis [3].

The selection of patients with HCC to undergo LR or LT relies mainly on morphological features assessed by liver dynamic imaging techniques, such as computed tomography and/or magnetic resonance. However, morphological features of HCC are poor surrogates of tumor aggressiveness. Smaller tumors showing microvascular invasion or poor histological differentiation may be more aggressive and prone to recur after the procedure [4,5]. A deeper understanding of HCC biology has allowed to identify non-invasive surrogate markers of tumor aggressiveness. Alpha-fetoprotein (AFP) is the most widely used in clinical practice and its combination with imaging-based has refined the selection of candidates for LT [5,6,7,8,9,10,11]. However, AFP has important limitations: 30–40% of HCCs do not elevate AFP, false positives may occur in acute and chronic hepatitis from different etiologies [12], and its optimal threshold has not been established [13].

Liquid biopsy allows for the detection of cancer byproducts in the bloodstream, such as circulating tumor cells (CTCs) [14]. The enumeration of CTCs is a non-invasive and reproducible technique which could dynamically monitor tumor activity [15]. In certain types of tumors, such as breast or lung cancer [16,17], an increased enumeration of CTCs has been associated with more aggressive tumor progression and mortality. Scientific evidence is scarce in HCC, but some preliminary studies have shown that CTC count before surgery is increased in patients with subsequent tumor recurrence [18,19,20,21]. Nevertheless, clearance kinetics of CTCs after surgery has not been investigated hitherto.

The primary aim of this pilot study was to evaluate the phenomenon of clearance of CTCs after surgery in patients with HCC, both in immunocompetent and in immunocompromised individuals. In addition, we analyzed the relationship between CTCs, HCC histological features, tumor recurrence, and overall survival.

## 2. Results

### 2.1. Study Population

A total of 41 patients with HCC were included, with a mean age of 58.7 ± 6.3 years old and a median follow-up after surgery of 30.5 months (IQR 16.3–34.9 months). There was a male predominance (82.9%). LR was performed in 10 patients (24.4%) and 31 patients (75.6%) underwent LT. The main etiology of liver disease was chronic hepatitis C (31.7%), followed by alcoholic liver disease (29.3%), and their combination (24.4%).

Baseline clinical characteristics and tumor features are shown in Table 1. Patients undergoing LR were older (63.2 vs. 57.2 years old, *p* = 0.058) and had received prior locoregional ablation therapy less frequently than patients in the LT group (20% vs. 90.3%, *p* = 0.001). Conversely, transplanted individuals had worse liver function (median MELD score 6 vs. 9; *p* = 0.002). There was a trend for increased diameter of the main nodule in resected patients (*p* = 0.051). An increased number of nodules was observed in patients undergoing LT, although without statistical significance (*p* = 0.092). There were no significant differences between resected and transplanted patients regarding moderate/poor histological differentiation (50% vs. 54.8%; *p* = 0.537) and microvascular invasion (10% vs. 19.5%, *p* = 0.444). Patients receiving LR and LT had no statistical differences in terms of tumor recurrence (40% vs. 16.1%, *p* = 0.127) and overall survival (87.1% vs. 90%; *p* = 0.647) during the follow-up period.

### 2.2. Circulating Tumor Cells’ Kinetics after Surgery

**Figure 1:**
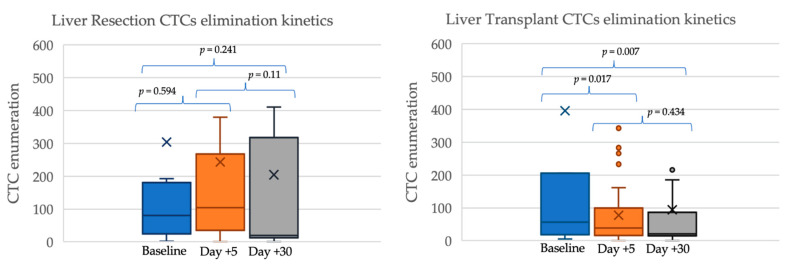
CTCs enumeration in patients with hepatocellular carcinoma undergoing LR (*n* = 10) or LT (*n* = 31).

The median enumeration of CTCs before surgery was 78 (IQR 23–200), with an early decline at post-operative day 5 (43 CTCs, IQR 17–120) and further stabilization until post-operative day 30 (20 CTCs, IQR 15–111). There were no statistical differences between patients undergoing LR and LT regarding CTC count at baseline (81 vs. 56; *p* = 0.99), at post-operative day 5 (103 vs. 38; *p* = 0.138), and at post-operative day 30 (20 vs. 21; *p* = 0.777—Figure 1). CTC clusters were also homogeneously distributed in LR and in LT patients: cluster detection was 50% vs. 61.3% at baseline (*p* = 0.394), 55.6% vs. 29% at day 5 (*p* = 0.142), and 30% vs. 35.5% at day 30 (*p* = 0.535). A progressive clearance of CTCs from baseline to post-operative day 30 was observed in the LT group (*p* = 0.007) but not in the LR group, in which the actual slope drifted towards a progressive, non-significant increase of CTC counts (*p* = 0.241). Trends in CTCs enumeration are shown in Figure 1 and Table 2.

The incomplete clearance of CTCs at post-operative day 30 occurred in 12 patients (38.7%) after LT and in 3 patients (30%) undergoing liver resection (*p* = 0.460). Interestingly, those patients with baseline CTC clusters were more likely to experience an incomplete CTC elimination at post-operative day 30 (54.2% vs. 11.8%, *p* = 0.005).

### 2.3. Prognostic Impact of CTCs

In the whole cohort, the relative decline of CTCs between baseline and post-operative day 30 was more pronounced in individuals with earlier stage tumors, although without statistical significance (Table 3): diameter of the main nodule < 30 mm vs. ≥30 mm (89.4% vs. 60.6% decline, respectively; *p* = 0.065), single nodule vs. multinodular HCC (72.6% vs. 62.8% decline; *p* = 0.552), and well-differentiated vs. moderate-poor differentiation (81.5% vs. 48% decline; *p* = 0.261). Patients with serum alpha-fetoprotein > 400 ng/mL experienced a steady increase of CTCs counts by 276% from baseline to post-operative day 5 (*p* = 0.04), although it was attenuated at post-operative day 30 (*p* = 0.349). Incomplete clearance of CTCs was less frequent in patients with microvascular invasion (29.4% vs. 71.4%), although without statistical significance (*p* = 0.079).

**Figure 2:**
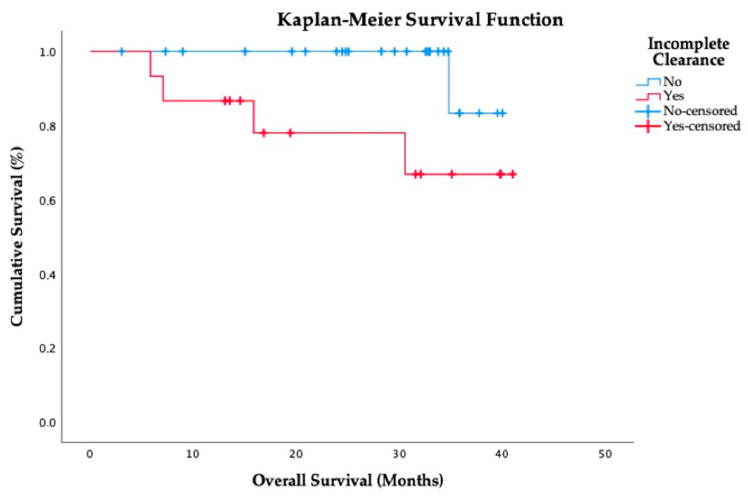
Kaplan–Meier curve showing the relationship between incomplete clearance of CTCs at post-operative day 30 and mortality.

During follow-up, 9 patients in the whole study population (22%) had HCC recurrence after surgery (4 patients from the LR group and 5 patients from the LT group). There were 5 deaths (12.2%), all of them being related with extrahepatic HCC recurrence. Neither preoperative CTCs enumeration nor an incomplete clearance at post-operative day 30 were associated with the risk of tumor recurrence (*p* = 0.91 and *p* = 0.43, respectively). However, the incomplete clearance of CTCs at post-operative day 30 was associated with increased mortality rates due to extrahepatic HCC recurrence (*p* = 0.038, Figure 2).

## 3. Discussion

This is the first study evaluating the clearance kinetics of CTCs in patients with HCC undergoing different surgical procedures. A relevant decline of CTC counts within the first month was observed in the vast majority of individuals. However, the clearance of CTCs was incomplete in some patients and this was associated with increased mortality, particularly due to aggressive extrahepatic tumor recurrence. On the other hand, an increased baseline enumeration of CTCs precluded their incomplete clearance at post-operative day 30, but it was not influenced by other widely known histological poor prognostic features, thus suggesting that CTCs enumeration may provide new information to identify patients with increased biological aggressiveness.

The enumeration of CTCs has become a subject of particular interest in the prediction of early tumor recurrence [14]. We evaluated the clearance of CTCs separately in immunocompetent patients, who underwent LR, and in immunocompromised individuals, who received a LT and immunosuppressive drugs immediately thereafter. We hypothesized that patients receiving immunosuppression would experience a slower decline of CTCs given that evading the immune system is one of the hallmarks of cancer. However, the observed effect was the opposite: immunocompetent patients undergoing LR had a slower decline. It may well be that the early decline depends mainly on the surgical technique. In such case, LT removes the whole liver, including other potential nascent tumors in the cirrhotic parenchyma, while in some patients undergoing LR, the preserved cirrhotic liver may include microscopic HCC foci therein, which could justify an incomplete clearance of CTCs and also the increased HCC recurrence rates as compared with LT. The effect of immunosuppression nowadays may be of less relevance for CTCs clearance early after transplantation, since minimization of calcineurin inhibitors is universally recommended [22]. The effect of a more prolonged exposure to calcineurin inhibitors on the clearance of CTCs requires further investigation.

CTCs detection techniques should ideally have high sensitivity and specificity, as well as acceptable reproducibility with low intra- and inter-observer variability. In addition, preserving the viability of the retrieved tumor cells would be of high interest for their ulterior characterization and culture [23,24], in order to analyze key mutations that confer resistance to systemic therapies [25]. There are many methodologies to identify and enumerate CTCs, and some of them have been previously tested in patients with HCC (Table 4). Hitherto, none of these methods can be considered the standard of care in HCC, and the combination of several methodologies would be needed to overcome the others’ disadvantages. Besides, few CTCs are actually viable and able to induce metastasis, as they would be a necessary but not a sufficient step for ulterior recurrence [26].

In our study, we relied on the expression of the epithelial adhesion molecule (EpCAM) for the detection of CTCs. EpCAM is a well-characterized surface marker of liver cancer cells, and the presence of EpCAM-positive CTCs has been previously associated with vascular invasion, increased serum AFP [21,34,35], more advanced HCC stage, and increased recurrence rates after surgery [21,36]. There are several approaches to enumerate EpCAM-positive CTCs, the most widely known being the CellSearch^TM^ system, which was used in some preliminary studies including HCC patients who underwent surgical resection of HCC [19,30,33]. However, the CellSearch^TM^ system has shown reduced sensitivity in patients with early HCC [37]. On the other hand, EpCAM could be lost during the epithelial to mesenchymal transition (EMT) process [38,39], as tumor undifferentiation leads epithelial cell lines to recover stem-cells’ characteristics, including the expression of other markers such as vimentin [40]. This theoretical EpCAM-negative cell population qualified for EMT and immune evasion [41] could have been overlooked in our study [42,43]. Future studies may consider a combination of markers for a wider recruitment of CTCs, which could provide a more accurate information to predict disease-free and overall survival in patients with HCC [20,29,32].

Some limitations of the present study are to be noted. There was a reduced sample size due to slow accrual, prolonged surveillance, and increased costs derived from CTCs analysis. Therefore, some observed trends without statistical significance need to be taken with caution, as they could be of clinical significance. In addition, we only explored the clearance kinetics of CTCs within the first post-operative month. A more prolonged surveillance could provide additional information about the interplay between CTCs and the immune system, particularly in LT individuals. Finally, results may have also been hampered by the restricted scope of our cell-processing methodology based exclusively on EpCAM.

Taking our results as a whole, we believe that liquid biopsy, and particularly the enumeration of CTCs, could become part of the armamentarium to diagnose HCC and to evaluate tumor response after different surgical and systemic therapies. A large multicenter consortium using cutting-edge technology of liquid biopsy could allow for a true personalized medicine in patients with HCC, thereby improving outcomes.

## 4. Materials and Methods

### 4.1. Study Design and Patient Inclusion

A consecutive cohort of patients with cirrhosis and HCC undergoing LR or LT were prospectively enrolled in this pilot study from September 2017 to May 2020. Exclusion criteria were as follows: age < 18 years old, human immunodeficiency virus infection, liver re-transplantation, combined organ transplantation, and early post-operative mortality (within the first month).

The selection of candidates for LR or LT was made by a multidisciplinary team aligning with the guidelines issued by the European Association for the Study of the Liver [2]. In patients enlisted for transplantation, locoregional bridging therapies, mainly transarterial chemoembolization, were used to prevent drop-out unless technically unfeasible. After LT, primary immunosuppression consisted in tacrolimus, mofetil mycophenolate (1–2 g/day), and tapering corticosteroids. Tacrolimus dose adjustments were made according to whole blood trough concentrations, which were aimed at 6–10 ng/mL within the first month, with a progressive reduction thereafter to obtain trough concentrations around 4 ng/mL in the long term. Basiliximab induction therapy was used in selected patients with pre-transplant renal impairment or with hepatic encephalopathy, in order to delay the introduction of tacrolimus until day five. Corticosteroids were progressively withdrawn during the first six months after LT.

Clinical visits were scheduled daily until hospital discharge, weekly until post-operative day 30, monthly within the first 3 months, every three months within the first year, and every six months thereafter, with additional visits whenever clinically indicated. HCC recurrence was assessed by using abdominal ultrasound and serum AFP every 3 months within the first year and every 6 months afterwards. Any suspicious liver nodule or AFP elevation were further evaluated by magnetic resonance or computed tomography. Imaging and histological criteria to diagnose HCC mirrored current international guidelines [2]. All study participants were required to provide written informed consent in order to participate in the study. The investigation protocol was in accordance with the Declaration of Helsinki (6th revision, 2008) and it was approved by the Andalusian Research Ethics Committee (code PI14/01469; ref. 239/2781).

### 4.2. Variables

The main outcome of the study was incomplete CTC clearance, as defined by a persistence of >30 CTCs at post-operative day 30. Secondary outcomes were HCC recurrence after surgery and overall mortality.

Demographic characteristics, etiology of liver disease, and radiological features of HCC before surgery were registered. Histological evaluation of explanted—or resected—liver specimens was systematically performed by an expert liver pathologist to determine the number of HCC nodules, the diameter of the largest nodule, total tumor diameter, histological differentiation according to the Edmonson scale [44], and macrovascular and microvascular invasion, as previously defined [4]. In patients undergoing LT, the primary immunosuppression protocol (drugs, dose, and trough concentrations) was also registered.

### 4.3. CTCs Isolation from Peripheral Blood

Peripheral blood samples were collected immediately before the surgical procedure, at post-operative day 5 (±1 day), and at post-operative day 30 (±2 days).

Blood samples were obtained by phlebotomy. The first 9 mL of blood were disregarded to avoid contamination with epithelial cells from the puncture site. BD Vacutainer^®^ spray-coated K_2_EDTA tubes were used to store samples at room temperature until analysis, which was performed within the first 24 h after the extraction.

For CTCs isolation, 7 mL of peripheral whole blood samples were processed by the CTC Enrichment Kit from the IsoFlux Liquid Biopsy System (Fluxion Biosciences, Alameda CA, USA), which is based on EpCAM expression. Firstly, we obtained the mononuclear cell fraction using Leucosep^®^ tubes (Greiner, Kremsmünster, Austria) and Ficoll-Paque Plus (GE Healthcare, Chicago, IL, USA), according to the manufacturer’s instructions. After centrifugation at 800× *g* for 15 min, the supernatant was decanted into a new 50 mL conical tube containing 10× CTL-Wash (CTL, Ohio, USA). The cellular suspension was centrifuged at 280× *g* for 10 min. The obtained cellular pellet was resuspended in a final volume of 880 µL with binding buffer (BB) and incubated, firstly, with 40 µL blocking solution for 5 min on ice, and afterwards, with 40 µL EpCAM-coated magnetic beads for 2h at 4 °C with rotation. Then, cells were transferred into a previously primed IsoFlux cartridge. When the CTC enrichment protocol was finished, the cartridge was immediately removed from the IsoFlux system and the magnetic beads were recovered with BB, using a micropipette and a magnet. Finally, immunocaptured cells were fixed in a 1.85% formaldehyde solution for 20 min and stored at 4 °C in BB until sample staining.

### 4.4. CTCs Staining and Analysis

Immunocaptured cells were stained with the CTC Enumeration Kit (Fluxion Biosciences, Alameda, CA, USA). All steps were performed at room temperature as recommended by the manufacturer. Briefly, cells isolated from peripheral blood were incubated with 10% normal donkey serum for 5 min, then with 1:100 rabbit anti-human CD45 primary antibody for 20 min, and finally with 1:200 donkey anti-rabbit IgG-Cy3 secondary antibody for 15 min in the darkness. Washing with 100 µL BB was performed before and after the last incubation. Then, cells were permeabilized with 0.2% Triton X-100 for 5 min and incubated with 1:10 mouse anti-human cytokeratin-FITC in the dark for 50 min. The cells were washed with 1X Hoechst in 0.02% Triton X-100 and resuspended in BB. In order to visualize the cells under a fluorescence microscope, the magnetic beads were deposited on a glass slide over a magnet. The excess of BB was removed, mounting method was added, and finally a glass coverslip was placed.

**Figure 3:**
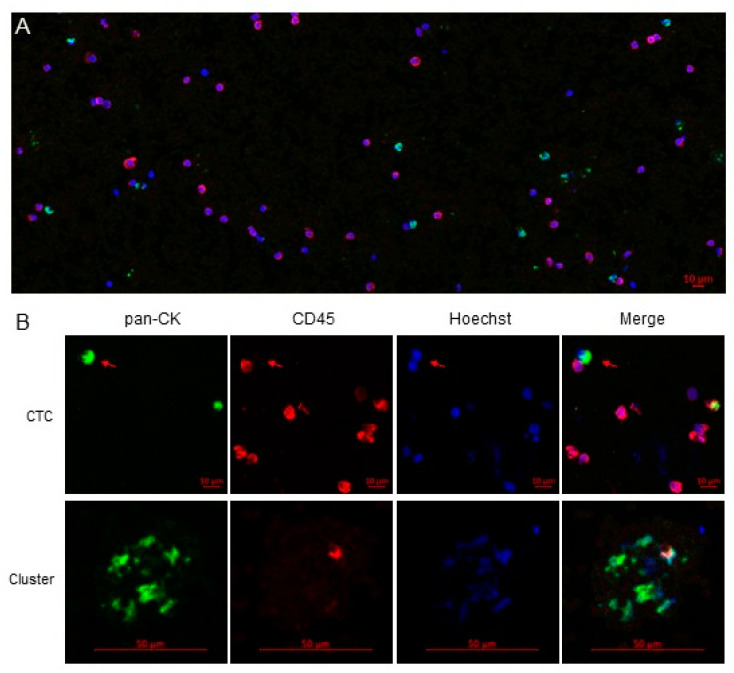
Analyses of CTCs isolated by the IsoFlux^TM^ System. (**A**) Fragment of a typical image obtained by confocal microscopy after scanning a sample stained with anti-human pan-CK and anti-human CD45 antibodies. Hoechst 33,342 was used to stain the cell nuclei. (**B**) Representative images of a typical CTC (nucleated, EpCAM^+^/CK^+^/CD45^−^) and a CTC cluster. Magnetic beads coated with anti-EpCAM used for CTCs enrichment can also be observed.

For the analysis, a single fluorescence image was obtained from the automatic scanning of each sample, using a spectral confocal microscope LSM710 (ZEISS, Oberkochen, Germany), which acquires optical sections with high contrast and resolution. Once the sample was delimited on the X and Y axes, we set four marks on the Z axis to have a complete and unequivocal image. The images were analyzed with a free microscope software ZEN Lite Blue/Black Edition from ZEISS Microscopy, identifying CTCs as those with an intact nucleated cell showing CK^+^/CD45^−^ (Figure 3). CTC clusters were defined as a groups of 3 or more CTCs aggregated in tight contact. In absence of previous studies, an incomplete clearance of CTCs was empirically defined as presence of >30 CTCs/sample at post-operative day 30. The cell counting was performed manually and independently by two trained members of the research group, who were blinded to the clinical data, based on the image generated therein. Any disagreement was resolved by a third investigator.

### 4.5. Statistical Analysis

Statistical analyses were performed using SPSS version 25.0 (SPSS Inc. Chicago, IL, USA). Variables were displayed in frequency tables or expressed as means and standard deviations, except for those with an asymmetric distribution, in which medians and interquartile ranges were used. Comparisons between groups were analyzed using Student’s *t* test, Wilcoxon test, Fisher test, Chi-square test, or Mann–Whitney’s *U* test, as appropriate. The correlation between incomplete CTC clearance and survival was analyzed using a Kaplan–Meier curve and the Log-rank test. All statistical analyses were two-tailed and a *p*-value of <0.05 was considered statistically significant.

## 5. Conclusions

The enumeration and characterization of CTCs is a rapidly evolving field which could reshape the current therapeutic algorithms of HCC. In the present study, we failed to demonstrate a link between baseline (EpCAM-positive) CTCs and HCC recurrence after LR or LT. However, the presence of clusters of CTCs before surgery was associated with an incomplete clearance of CTCs at post-operative day 30, which in turn predicted mortality due to extrahepatic recurrence of HCC. The phenomenon of incomplete clearance of CTCs after potentially curative surgery should be further investigated as these patients could probably benefit from a closer surveillance and adjuvant systemic therapies.

## Figures and Tables

**Table 1:** Baseline characteristics of patients undergoing curative treatment.

Variable	Resection (*n* = 10)	Transplantation (*n* = 31)	*p*
Age (years)	63.2 ± 8.5	57.2 ± 4.7	0.058
Sex (men), *n* (%)	8 (80%)	26 (83.9%)	0.556
Hepatitis B, *n* (%)	2 (20%)	4 (12.9%)	0.458
Hepatitis C, *n* (%)	4 (40%)	19 (61.3%)	0.208
Alcoholic liver disease, *n* (%)	4 (40%)	18 (58.1%)	0.264
Portal Hypertension, *n* (%)	1 (10%)	24 (77.4%)	0.001
Child-Pugh score	5 (5–5)	6 (5–7)	0.003
MELD score	6 (6–7)	9 (7–12)	0.002
Number of nodules, *n* (%)			
Single nodule	9 (90%)	19 (61.3%)	0.092
Multinodular	1 (10%)	12 (38.7%)	
Main nodule diameter (mm)	34.4 ± 22.8	22.1 ± 14.4	0.051
Histological differentiation (moderate–poor), *n* (%)	5 (50%)	17 (54.8%)	0.537
Microvascular invasion, *n* (%)	1 (10%)	6 (19.5%)	0.444
Baseline AFP (ng/mL)	8.33	4.55	0.421
	(IQR 1.9–186.7)	(IQR 2–16.2)	
Bridging locoregional therapy, *n* (%)	2 (20%)	28 (90.3%)	0.001
Primary immunosuppression			
Tacrolimus, *n* (%)	-	31 (100%)	-
Mycophenolate, *n* (%)	-	31 (100%)	-
Everolimus, *n* (%)	-	1 (3.2%)	-
Basiliximab induction, *n* (%)	-	3 (9.7%)	-
Tacrolimus trough levels in the first month (ng/mL)	-	6.9 ± 1.7	-
Median follow-up after surgery (months)	33 (IQR 30–37)	25 (IQR 15–34)	0.075
HCC recurrence after surgery, *n* (%)	4 (40%)	5 (16.1%)	0.127
Mortality, *n* (%)	1 (10%)	4 (12.9%)	0.647

**Table 2:** CTC clusters and total enumeration. Median and interquartile range are presented.

Variable	Resection (*n* = 10)	Transplant (*n* = 31)	*p*
Baseline CTC enumeration	81 (24–178)	56 (18–204)	0.990
Baseline CTC clusters	1 (0–2)	1 (0–5)	0.764
CTC enumeration day +5	103 (34.5–258)	38 (16–99)	0.138
CTC clusters day +5	1 (0–13)	0 (0–1)	0.114
CTC enumeration day +30	20 (13–311)	21 (15–85)	0.777
CTC clusters day +30	0 (0–5)	0 (0–1)	0.846
Incomplete clearance	3 (30%)	12 (38.7%)	0.460

**Table 3:** Relative delta changes of CTCs enumeration and clusters from baseline to post-operative day 30 according to clinical features.

Variable		Relative Change of CTCs	*p*	Increase in CTC Clusters	*p*
Sex	Male (*n* = 34)	−71.34% (−91.79–−6.25%)	0.058	11.8%	0.458
	Female (*n* = 7)	+33.3% (−70.9–382.85%)		0%	
Age	<60 (*n* = 26)	−65.3% (−89.9–60.76%)	0.799	15.4%	0.148
	≥60 (*n* = 15)	−74.36% (−91.25–33.33%)		0%	
Type of surgery	Resection (*n* = 10)	−71.34% (−84.9–−20.2%)	0.846	20%	0.245
	Transplant (*n* = 31)	−62.8% (−91.25–60%)		6.5%	
AFP (ng/mL)	<400 (*n* = 34)	−69.6% (−91.8–40.76%)	0.349	8.8%	0.838
	≥400 (*n* = 2)	+21.5% (−48–21.5%)		0%	
Number of nodules	Single nodule (*n* = 28)	−72.6% (−93.19–68.2%)	0.552	10.7%	0.623
	Multinodular (*n* = 13)	−62.8% (−83.9–−2.3%)		7.7%	
Dominant nodule size (mm)	<30 (*n* = 30)	−60.6% (−86.07–75.16%)	0.065	10%	0.712
	≥30 (*n*=11)	−89.4% (−93.26–−48%)		9.1%	
Histological Differentiation	Well (*n* = 22)	−81.46% (−91.8–62.5%)	0.261	9.1%	0.639
	Moderate-poor (*n* = 19)	−48% (−85.9–33.3%)		10.5%	
Microvascular invasion	Absent (*n* = 34)	−68.1% (−88–12.5%)	0.799	5.9%	0.128
	Present (*n* = 7)	−62.85% (−94.11–90.9%)		28.6%	

**Table 4:** Studies evaluating CTCs in patients with HCC undergoing liver resection (LR) or liver transplantation (LT).

Reference	*n*	Therapy	CTCs Technique	CTCs Threshold	Time-Point Analysis	Prognostic Impact
Sun et al. [19]	123	LR	CellSearch^®^	≥2/7.5 mL	Baseline and at 1 month	Increased tumor recurrence
Ramirez et al. [27]	24	LT	Isoflux^®^	Not defined	Baseline, at 1 month and at 6 months	No association with outcomes
Ou et al. [20]	165	LR	CanPatrol^®^	≥2/5 mL	Baseline	Shorter overall survival
Xue et al. [18]	30	LT	iFISH + CellSearch^®^	≥5/7.5 mL	Baseline and at 3 months	Reduced recurrence-free survival
Ye et al. [28]	42	LR	CanPatrol^®^	≥5/5 mL	Baseline and at 1 month	Reduced recurrence-free survival
Wang et al. [29]	47	LT	CanPatrol^®^	Not defined	Baseline and at 1 month	No association with outcomes
Yu et al. [30]	139	LR	CellSearch^®^	≥2/7.5 mL	Baseline and at 3 days	Reduced overall survival
Chen et al. [31]	143	LR	CanPatrol^®^	Not defined	Baseline and after therapy	No association with outcomes
Chen et al. [32]	50	LT	Negative enrichment + imFISH	≥2/3.2 mL	Baseline	Increased recurrence and reduced survival
Sun et al. [33]	197	LR	CellSearch^®^	≥3/7.5 mL	Baseline and at 1 month	Reduced survival

CellSearch: Menarini, Florence, Italy; Isoflux: Fluxion Biosciences, Alameda, CA, USA; CanPatrol: SurExam, Guangzhou, China.

## Data Availability

Data available on request due to restrictions (sharing data was not planned before the ethics committee approval of the study).
